# Oncolytic Virotherapy for High-Grade Glioma and Current Evidence and Factors to Consider for Incorporation into Clinical Practice

**DOI:** 10.3390/pathogens12070861

**Published:** 2023-06-22

**Authors:** Sauson Soldozy, Daniel G. Eichberg, Alexis A. Morell, Evan Luther, Victor M. Lu, Dominique M. O. Higgins, Nitesh V. Patel, Ashish H. Shah, Simon J. Hanft, Ricardo J. Komotar, Michael E. Ivan

**Affiliations:** 1 Department of Neurosurgery, Westchester Medical Center, New York Medical College, 100 Woods Road, Valhalla, New York, NY 10595, USA; 2 Department of Neurological Surgery, University of Miami, 1295 NW 14th St, Miami, FL 33125, USA; 3 Department of Neurosurgery, University of North Carolina Medical Center, 101 Manning Dr, Chapel Hill, NC 27514, USA; 4 Department of Neurosurgery, Hackensack Meridian School of Medicine, Hackensack Meridian Health—Jersey Shore University Medical Center, Nutley, NJ 07110, USA

**Keywords:** oncolytic virotherapy, glioma, virus therapy, glioblastoma, brain tumor

## Abstract

Brain tumor incidence is on the rise, and glioblastoma comprises the majority of primary tumors. Despite maximal safe resection and adjuvant chemoradiation, median survival for high-grade glioma remains poor. For this reason, it is important to develop and incorporate new treatment strategies. Oncolytic virotherapy has emerged as a viable new therapeutic entity to fill this gap. Preclinical research has shown oncolytic virotherapy to be a robust and effective treatment option for brain tumors, and clinical trials for both adult and pediatric high-grade glioma are underway. The unique and protected environment of the nervous system, in part due to the blood–brain barrier, prevents traditional systemic therapies from achieving adequate penetration. Brain tumors are also heterogenous in nature due to their diverse molecular profiles, further complicating systemic treatment efforts. Oncolytic viruses may serve to fill this gap in brain tumor treatment given their amenability to genetic modification and ability to target unique tumor epitopes. In addition, direct inoculation of the oncolytic virus agent to the tumor bed following surgical resection absolves risk of systemic side effects and ensures adequate delivery. As virotherapy transitions from bench to bedside, it is important to discuss factors to make this transition more seamless. In this article, we describe the current clinical evidence as it pertains to oncolytic virotherapy and the treatment of brain tumors as well as factors to consider for its incorporation into neurosurgical workflow.

## 1. Introduction

The incidence of malignant brain tumors is rising, with the most common primary brain malignancy being glioblastoma (GBM) [1,2,3,4,5]. Despite decades of innovation, the current treatment paradigm of triple therapy, including maximal safe resection, radiation, and chemotherapy, are inadequate and survival remains poor [6]. These treatment modalities are limited in part by the immune-privileged status of the central nervous system (CNS). Additionally, recent findings show direct neuronal synaptic communication with tumor cells triggers generation of tumor microtubules and tumor cell locomotion/translocation, conferring the characteristic infiltrative nature of GBM and further complicating treatment [7].

A newly emerging therapeutic tool in cancer treatment, oncolytic virotherapy (OV), serves as a potential means to bypass current limitations to GBM treatment. Combining direct tumor cell lysis with local immune stimulation, OV induces tumor cell death through various mechanisms [8,9]. Transgenic editing and virus pathogenicity enables highly selective targeting of tumor cells while sparing normal tissues, making OV highly favorable in this regard [10,11,12,13,14,15,16,17]. Viral vectors can also be modified to induce cellular transcription and translation of desired proteins, including enzymes that activate non-toxic chemotherapeutic prodrugs into their cytotoxic metabolites. While traditionally toxic to healthy cells, this allows for delayed activation of prodrugs until reaching the tumor microenvironment [18,19].

Clinical trials for both adult and pediatric high-grade tumors are either recently completed or ongoing, and preliminary results are promising [9,17,20].

As OV continues to gain traction in brain tumor research, it is important to consider barriers to clinical entry to enable seamless transition to the bedside. The first question is which viral motif to implement as current trials employ a large variety of different viral vectors [9,17]. The next question is delivery method, with direct tumor inoculation following surgical resection appearing to be the least disruptive to the current neurosurgical work-flow. Intra-arterial infusion is another newly emerging avenue for OV delivery [21]. This raises the question of how OV may fit into current triple therapy paradigms as data regarding this remains limited. Additionally, the need for subsequent repeat dosing for OV remains to be elucidated; however, this may be achieved through minimally invasive means such as stereotactic or convection-enhanced delivery.

In this review, the authors discuss the current state of oncolytic virotherapy for high-grade glioma along with considerations for its incorporation into clinical brain tumor practice.

## 2. Origin of Oncolytic Viruses

The properties of oncolytic viruses were observed prior to the characterization of viruses themselves. Early anecdotal accounts can be found in the literature as early as 1904 describing cancer patients who went into remission following an infection with influenza or measles virus [22,23,24,25]. Viral particles were first observed with electron microscopy in 1939, and the rise of tissue culturing allowing for ex vivo virus propagation greatly increased understanding of viruses in the 1950s and 1960s [26,27,28,29]. Many clinical trials were initiated between 1950 and 1970, however poor study designs and lack of quality control led to adverse events and limits interpretation of these data; additionally, the advent of chemotherapy led to a decline in interest in oncolytic viruses [30,31]. Greater understanding of the viral genome and viral replication, along with the rise of virus-based gene therapy in the 1990s, sparked renewed interested [27,32].

## 3. Current Evidence

### 3.1. Adult High-Grade Glioma

The last decade has seen an increasing trend in OV clinical trials for adult high-grade glioma [17]. The majority of trials are phase I with the primary outcome of interest being safety and toxicity. The most commonly utilized viral vector is adenovirus, followed by herpes simplex virus [15,17,33]. Many viral variants for adenovirus have been developed, with the Delta-24-RDG variant advancing the furthest in clinical trials [34]. In a phase I trial of 37 patients with recurrent malignant glioma, Lang et al. [35] demonstrated for the first time in humans the histopathologic evidence of direct virus-induced oncolysis and virus-mediated cytotoxic immune response. Specimens obtained greater than one month after viral injection did not show evidence of virus; however, clinical responses did not occur until >3 months post-injection, suggesting a secondary delayed viral induced antitumor immune response is at play [35]. There is ongoing study regarding the most optimum adenoviral serotype, with majority of studies utilizing adenovirus 5 (HAdV-C5), specifically with respect to tumor cell selectivity [33]. Coxsackie adenovirus receptor (CAR), and CD46, are adenovirus entry receptors that are highly expressed in both healthy brain and GBM; therefore, transgenic editing should be explored to further augment adenoviral specificity for tumor cells [33].

**Figure 1:**
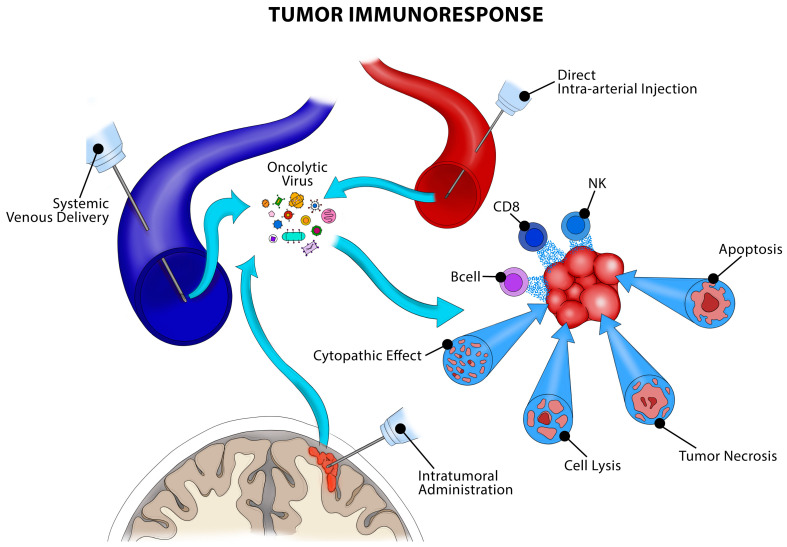
Oncolytic virotherapy can be administered through several routes. The most common is systemic delivery through venous administration. While straightforward, intravenous delivery confers the disadvantage of possible systemic toxicity and potentially reduced bioavailability when compared to other delivery methods. Another option is to inject inoculated virus into the tumor as well as the tumor resection cavity. This enables direct delivery to the tumor tissue during or immediately after surgical resection, allowing localized viral replication and tumor cell destruction. However, the degree of viral penetration into adjacent tumor cells is unclear as injected virus may remain focused within the injection site. Alternatively, selective angiographic catheterization of feeding tumor vessels allows for intra-arterial delivery of oncolytic viruses. Regardless of delivery method, a similar tumor immunoresponse is triggered including tumor cell lysis and recruitment of immune cells such as natural killer (NK) cells, Bcells, and CD8 cells. Artwork courtesy of Roberto C. Suazo, Medical Illustrator and Graphic Design Project Manager for the Department of Neurological Surgery, University of Miami.

It is well known that high-grade gliomas produce immunosuppressive tumor microenvironments in part due to a phenomenon known as T-cell exhaustion mediated by tumor-associated macrophages/microglia (TAMs) and myeloid-derived suppressor cells (MDSCs) [15,36,37,38,39,40]. Tumor-associated macropahges/microglia (TAMs) make up to 50% of the tumor mass and are largely derived from native brain microglia as opposed to peripherally derived macrophages; however, this is dependent on the mutation status (IDH1 versus wild-type) [41]. It is known that OV results in further recruitment of microglia and peripheral macrophages; however, the interaction between OV and these immune cells is not fully understood. In fact, both an anti-viral response and anti-tumor response may be triggered [41]. This suggests that there may be a different tumor response to OV depending on mutation status; however, further research in this domain is necessary. Other sources of immunosuppression include adjuvant therapies such as chemotherapy, radiation, and corticosteroid administration. Despite these hindrances, OV is still able to induce a local immune response by recruiting local T lymphocytes, natural killer cells, and macrophages among other immune cells (Figure 1) [15,35]. Therefore, in addition to its lytic properties, virotherapy appears to induce a robust local immune response that circumvents immunosuppression induced by high-grade gliomas. This has been shown in preclinical studies and was confirmed in humans by Lang et al. [35]. Anti-PD-1 antibodies in combination with Delta-24-RGD are theorized to augment this effect (NCT02798406, results pending). Another adenovirus variant, Delta-24-RGDOX, has been engineered to encode for mouse OX40 L, a ligand for the T-cell-activating receptor OX40 found on T cells. This has been shown in murine models to further enhance intratumoral T-cell infiltration and is currently in the recruiting stage of a phase I trial (NCT04214392). These studies demonstrate that isolated OV may be insufficient to provide adequate infiltration and bypass anti-viral immune response; however, when administered in conjunction with anti-tumor molecules, this may augment the lytic and immunogenic response triggered by the virus.

In addition to the intrinsic immunogenic and oncolytic properties of the aforementioned viruses, OV can be used to modify the tumor microenvironment. Cellular and non-cellular components of the tumor microenvironment are known to interact with and promote tumor cell growth [7,40]. Hyaluronan or hyaluronic acid (HA) is a component of the extracellular matrix that augments tumor proliferation via binding of tumor cell surface receptors including CD44 and receptor for hyaluronan-mediated motility (RHAMM). In addition to its tumorigenic properties, HA, specifically the high-molecular weight variant, has been shown to inhibit virus-induced NF-kB signaling in macrophages, reducing the efficacy of OV agents [40]. In a modified Delta-24-RGD virus containing the SAPM1 gene encoding human hyaluronidase PH20, Kiyokawa et al. demonstrated decreased levels of HA within the GBM extracellular matrix in orthotopic 005 GBM murine models; consequently, this mediated increased tumor infiltration of CD3, CD8, tumor-associated macrophages, and PD-L1, resulting in prolonged animal survival [40]. Survival was further prolonged with the addition of anti-PD-1 antibody.

Another advantage of virotherapy is the ability to enhance existing therapies. For instance, chimeric antigen receptor T (CAR-T) cells have demonstrated excellent anti-tumor effects in hematologic malignancies, resulting in FDA approval; however, less efficacy has been observed in GBM due to poor infiltration [42,43]. Given that CAR-T cells rely on chemokine receptors and matched chemokines on tumor cells for effective infiltration, Wang et al. developed an oncolytic adenovirus containing CXC ligand 11 (CXCL11) [43]. Chemokine CXCL11 levels were greatly increased, resulting in enhanced CAR-T cell infiltration within the tumor microenvironment and greater recruitment of immune cells [43]. This highlights the significant advantage of OV as more clinical data emerges, and variations of existing viral vectors can be quickly developed to hone in on and augment its immune mediated anti-tumor response. Given the molecular diversity of brain tumors, this opens the door for personalized viral vectors tailored to a patient’s unique disease presentation as well.

While the current literature demonstrates that OV prolongs survival in animal models, and phase I trials suggest OV to be safe, evidence for its efficacy in prolonging survival in human subjects remains more limited. A systematic review by Lu et al. [17]. reported a median progression free survival of 3 months (range 1–8 months) across 8 trials, with a median overall survival from first viral dose to be 15 months (range 11–32 months) across 12 studies. It is important to note that all studies evaluated patients with recurrent GBM; therefore, time from initial diagnosis should be added to the reported overall survival values to gain a true sense of OV efficacy in these cohorts [17]. No trials studied primary high-grade glioma alone upon initial diagnosis, and it would be interesting to evaluate the efficacy of OV earlier in a patient’s disease course prior to treatment and subsequent tumor recurrence. There are reports of patients surviving many years (up to 14 years) after virus inoculation; however, this is limited to isolated cases [35,44]. In general, evidence for OV efficacy in adults is limited by small sample sizes with most studies enrolling less than 50 patients [15,17]. The large heterogeneity across trials, specifically regarding the use of different viral vectors, prevents meaningful comparisons from being drawn across studies. To date, no studies directly comparing different viral vectors have been conducted. A standardized viral vector and treatment paradigm, multi-center design, and larger patient recruitment is necessary to better elucidate the true efficacy of OV for glioblastoma in the adult population.

### 3.2. Pediatric High-Grade Glioma

Oncolytic virotherapy is also being explored for pediatric high-grade glioma. Similar to adults, prognosis for these lesions remains poor with median overall survival of 5.6 months despite radiation and chemotherapy [45]. Evidence for OV and pediatric high-grade glioma is largely preclinical; however, several clinical trials are in progress or have recently published data [9,11].

An open-label phase I non-randomized trial (NCT02457845) utilizing HSV G207 recently published its findings in 12 pediatric (age 3 to 18 years) patients with supratentorial HGG [11]. Of these tumors, there were ten glioblastomas, one anaplastic astrocytoma, and one unspecified high-grade glioma. Patients with evidence of tumor progression after surgery, radiation, or chemotherapy were eligible for inclusion. Subjects underwent placement of up to four intratumoral catheters followed by inoculation of the virus for a 6 h period. In six patients, gross tumor volume plus 2 mm margins received 5 Gy of radiation 24 h after inoculation. No dose-limiting or adverse events were observed, and a median overall survival of 12.2 months (95% CI 8.0–16.4) was reported. Four patients remained alive at 18 months post-injection. Biopsy samples taken 9 months post-injection revealed infiltration of CD8+ T cells, indicating local immune response secondary to viral injection [11]. These promising results have led to the planning of a phase II study.

In a preclinical study, Martínez-Vélez et al. [46] showed promising results with Delta-24-RGD in murine models of both pediatric HGG and diffuse intrinsic pontine gliomas (DIPG), demonstrating both robust local lymphocytic infiltration as well as prolonged survival in both cell lines. This triggered the initiation of a clinical trial (NCT03178032) by Pérez-Larraya et al. [47], which was recently published. In this trial, the authors utilized DNX-2401, an oncolytic adenovirus, in a series of eight patients (3–18 years old) with newly diagnosed, biopsy-confirmed DIPG. Median overall survival was 11.2 months [48]. Other clinical trials utilizing different vectors including oncolytic polio/rhinovirus, reovirus, and adenovirus are in progress [9].

Similar to adult cohorts, OV was reserved only in patients with later stage disease after failure of traditional therapies rather than earlier in the disease course. It remains unclear in both adults and children how earlier administration of OV may impact efficacy.

### 3.3. Methods of Virus Delivery

To date, there have been no reports of serious adverse events related to OV administration including encephalitis or death [15]. Direct intratumoral injection and viral injection into the resection cavity are the most commonly employed delivery methods. The existing evidence suggests patients tolerate these delivery methods well without signs of toxicity. However, disadvantages of intratumoral injection include limited viral distribution and backflow [34]. In fact, lack of virus detection after intratumoral injection has been reported [35]. This suggests injection cannulas placed at the center of the tumor may limit virus spread to the tumor edges [34,35]. Convection-enhanced delivery may serve to circumvent this issue, and a phase I trial utilizing this delivery method is in the recruitment stage (NCT04214392). Data for systemic administration is more sparce, and delivery via this method is limited by the blood–brain barrier and blood–tumor barrier. There is also the concern of systematic side effects; however, a recent phase II trial showed no adverse events [49]. Endovascular intra-arterial delivery of OVs is being explored as well, conferring the advantage of a 100-fold increase in concentration of therapeutic agent delivery in the brain [21,34]. Chen et al. recently merged magnetic resonance angiographic perfusion imaging with super-selective microcatheter injections to precisely identify the feeding arterial pedicle to GBM, enabling precision delivery of Delta-24-RGD (MSC-D24) [21]. Each aforementioned method conferes different advantages and disadvantages, and perhaps a combination of administration routes may be warrented to maximize viral delivery.

## 4. From Bench to Bedside: Factors to Consider

With oncolytic virotherapy transitioning from bench to bedside, it is important to discuss practical considerations from a clinical perspective. From a patient safety standpoint, it is of utmost importance to confirm minimal to no harm with OV administration as this remains a major clinical barrier of entry. The current data available suggest OV inoculation to be safe as no adverse reactions have been reported. In fact, OV appears to be better tolerated than traditional chemotherapies. Future studies should continue to confirm lack of toxicity in larger scale cohorts so that clinicians can be reassured OV administration carries a low-risk profile. To date, no single oncolytic virus has been sufficiently studied, with current clinical trials employing a large variety of viral vectors. Adenovirus and herpes virus remain the most commonly studied vectors, and preliminary clinical data is promising regarding safety and efficacy; however, further studies are needed to validate these findings. At this time, the question that still remains is which oncolytic virus to utilize in patients with glioblastoma. As more evidence for its safety emerges, clinicians can be reassured OV administration is without excessive harm.

Many avenues for OV administration exist, including systemic, super-selective intra-arterial, intratumoral, and resection cavity injections (Figure 1). The present data provides most evidence for intratumoral injection and resection cavity injection. The questions that remain are how much virus to inject and the need for subsequent dosing. It appears that placement of intratumoral catheters and prolonged inoculation over the course of several hours ensures adequate viral delivery versus a single stereotactic injection. Regarding repeat dosing, tumor samples collected many months post-injection show persistent local tumor immune response; however, in some patients, no immune response was observed. Repeat inoculation may be employed on a case-by-case basis; however, there is minimal data regarding this [50]. Viral injection after surgery to the resection cavity ensures adequate delivery to the tumor margins versus intratumoral injection, and present data has not shown negative sequelae from this method [9,11,20]. For patients undergoing maximally safe resection, injecting OV to the tumor bed following surgery is minimally disruptive to the neurosurgeon’s current clinical work-flow. The present data also remains limited regarding concomitant radiation, chemotherapy, and corticosteroid treatment. It is unclear what interactions, if any, may exist. While preclinical data suggests there to be a synergistic effect, clinical data in this regard is lacking. As it stands, virotherapy is emerging as a promising treatment option for high-grade glioma; however, further validation in the form of largescale multi-center clinical trials at high-volume brain tumor centers is necessary.

## 5. Conclusions

Oncolytic virotherapy for high-grade gliomas has made major strides in the last decade. Virotherapy is currently in the transition phase from bench to bedside, with preliminary phase I data showing oncolytic virotherapy to be well tolerated in patients receiving direct tumor and tumor bed injections. To date, no serious adverse reactions including encephalitis or death from virus inoculation have been reported. As more trials are completed, the toxicity profile or lack thereof will continue to be better established. Data regarding the efficacy of oncolytic virotherapy and prolongation of survival is less robust and difficult to generalize given the small sample sizes and large heterogeneity across clinical trials. Currently, there lacks a standard viral treatment paradigm as many different viral vectors are currently being explored in the literature. No single virus has been studied sufficiently enough at this time, but as more data becomes available it is expected that the scope of investigation will narrow down to either a single or few agents. From a clinical perspective, the incorporation of oncolytic virotherapy to neurosurgical practice will likely be minimally disruptive as inoculation can take place at the time of surgery. However, the questions that still remain are which viral agent to use, what dose to inject, the need for subsequent dosing, and what interactions exist with existing treatments including radiation, chemotherapy, and corticosteroids. While the field of oncolytic virotherapy for high-grade gliomas remains in its nascency, the existing data is promising, and, with further validation and larger-scale studies, it may serve to be a promising new therapeutic option.

## Data Availability

No applicable.

## References

[1] Zada G., Bond A.E., Wang Y.P., Giannotta S.L., Deapen D. (2012). Incidence trends in the anatomic location of primary malignant brain tumors in the United States: 1992–2006. World Neurosurg..

[2] Dobes M., Khurana V.G., Shadbolt B., Jain S., Smith S.F., Smee R., Dexter M., Cook R. (2011). Increasing incidence of glioblastoma multiforme and meningioma, and decreasing incidence of Schwannoma (2000–2008): Findings of a multicenter Australian study. Surg. Neurol. Int..

[3] Dobes M., Shadbolt B., Khurana V.G., Jain S., Smith S.F., Smee R., Dexter M., Cook R. (2011). A multicenter study of primary brain tumor incidence in Australia (2000–2008). Neuro-Oncology.

[4] Philips A., Henshaw D.L., Lamburn G., O’Carroll M.J. (2018). Brain tumours: Rise in glioblastoma multiforme incidence in England 1995–2015 suggests an adverse environmental or lifestyle factor. J. Environ. Public Health.

[5] Tabouret E., Chinot O., Metellus P., Tallet A., Viens P., Goncalves A. (2012). Recent trends in epidemiology of brain metastases: An overview. Anticancer Res..

[6] Ostrom Q.T., Cioffi G., Gittleman H., Patil N., Waite K., Kruchko C., Barnholtz-Sloan J.S. (2019). CBTRUS Statistical Report: Primary Brain and Other Central Nervous System Tumors Diagnosed in the United States in 2012–2016. Neuro Oncol..

[7] Venkataramani V., Yang Y., Schubert M.C., Reyhan E., Tetzlaff S.K., Wissmann N., Botz M., Soyka S.J., Beretta C.A., Pramatarov R.L. (2022). Glioblastoma hijacks neuronal mechanisms for brain invasion. Cell.

[8] Lawler S.E., Speranza M.C., Cho C.F., Chiocca E.A. (2017). Oncolytic Viruses in Cancer Treatment: A Review. JAMA Oncol..

[9] Soldozy S., Skaff A., Soldozy K., Sokolowski J.D., Norat P., Yagmurlu K., Sharifi K.A., Tvrdik P., Park M.S., Kalani M.Y.S. (2020). From Bench to Bedside, the Current State of Oncolytic Virotherapy in Pediatric Glioma. Neurosurgery.

[10] Iorgulescu J.B., Reardon D.A., Chiocca E.A., Wu C.J. (2018). Immunotherapy for glioblastoma: Going viral. Nat. Med..

[11] Friedman G.K., Johnston J.M., Bag A.K., Bernstock J.D., Li R., Aban I., Kachurak K., Nan L., Kang K.D., Totsch S. (2021). Oncolytic HSV-1 G207 Immunovirotherapy for Pediatric High-Grade Gliomas. N. Engl. J. Med..

[12] Fares J., Ahmed A.U., Ulasov I.V., Sonabend A.M., Miska J., Lee-Chang C., Balyasnikova I.V., Chandler J.P., Portnow J., Tate M.C. (2021). Neural stem cell delivery of an oncolytic adenovirus in newly diagnosed malignant glioma: A first-in-human, phase 1, dose-escalation trial. Lancet Oncol..

[13] Nigim F., Esaki S., Hood M., Lelic N., James M.F., Ramesh V., Stemmer-Rachamimov A., Cahill D.P., Brastianos P.K., Rabkin S.D. (2016). A new patient-derived orthotopic malignant meningioma model treated with oncolytic herpes simplex virus. Neuro Oncol..

[14] Chastkofsky M.I., Pituch K.C., Katagi H., Zannikou M., Ilut L., Xiao T., Han Y., Sonabend A.M., Curiel D.T., Bonner E.R. (2021). Mesenchymal Stem Cells Successfully Deliver Oncolytic Virotherapy to Diffuse Intrinsic Pontine Glioma. Clin. Cancer Res..

[15] Wang J.L., Scheitler K.M., Wenger N.M., Elder J.B. (2021). Viral therapies for glioblastoma and high-grade gliomas in adults: A systematic review. Neurosurg. Focus.

[16] Omar N.B., Bentley R.T., Crossman D.K., Foote J.B., Koehler J.W., Markert J.M., Platt S.R., Rissi D.R., Shores A., Sorjonen D. (2021). Safety and interim survival data after intracranial administration of M032, a genetically engineered oncolytic HSV-1 expressing IL-12, in pet dogs with sporadic gliomas. Neurosurg. Focus.

[17] Lu V.M., Shah A.H., Vallejo F.A., Eichberg D.G., Luther E.M., Shah S.S., Komotar R.J., Ivan M.E. (2021). Clinical trials using oncolytic viral therapy to treat adult glioblastoma: A progress report. Neurosurg. Focus.

[18] Studebaker A.W., Hutzen B.J., Pierson C.R., Haworth K.B., Cripe T.P., Jackson E.M., Leonard J.R. (2017). Oncolytic Herpes Virus rRp450 Shows Efficacy in Orthotopic Xenograft Group 3/4 Medulloblastomas and Atypical Teratoid/Rhabdoid Tumors. Mol. Ther. Oncolytics.

[19] Soldozy S., Mulligan K.M., Zheng D.X., Levoska M.A., Cullison C.R., Elarjani T., Eichberg D.G., Ampie L.E., Shah A.H., Yagmurlu K. (2021). Oncolytic Virotherapy for Melanoma Brain Metastases, a Potential New Treatment Paradigm?. Brain Sci..

[20] Carpenter A.B., Carpenter A.M., Aiken R., Hanft S. (2021). Oncolytic virus in gliomas: A review of human clinical investigations. Ann. Oncol..

[21] Chen S.R., Chen M.M., Ene C., Lang F.F., Kan P. (2022). Perfusion-guided endovascular super-selective intra-arterial infusion for treatment of malignant brain tumors. J. Neurointerv. Surg..

[22] Dock G. (1904). The influence of complicating diseases upon leukaemia. Am. J. Med. Sci..

[23] Taqi A., Abdurrahman M., Yakubu A., Fleming A. (1981). Regression of Hodgkin’s disease after measles. Lancet.

[24] Bluming A., Ziegler J. (1971). Regression of Burkitt’s lymphoma in association with measles infection. Lancet.

[25] Pack G.T. (1950). Note on the experimental use of rabies vaccine for melanomatosis. AMA Arch. Dermatol. Syphilol..

[26] Kausche G.A., Pfankuch E., Ruska H. (1939). Die Sichtbarmachung von pflanzlichem virus im Übermikroskop. Naturwissenschaften.

[27] Kelly E., Russell S.J. (2007). History of oncolytic viruses: Genesis to genetic engineering. Mol. Ther..

[28] Gey G. (1952). Tissue culture studies of the proliferative capacity of cervical carcinoma and normal epithelium. Cancer Res..

[29] Weller T.H., Robbins F.C., Enders J.F. (1949). Cultivation of poliomyelitis virus in cultures of human foreskin and embryonic tissues. Proc. Soc. Exp. Biol. Med..

[30] Hammill A.M., Conner J., Cripe T.P. (2010). Oncolytic virotherapy reaches adolescence. Pediatr. Blood Cancer.

[31] Larson C., Oronsky B., Scicinski J., Fanger G.R., Stirn M., Oronsky A., Reid T.R. (2015). Going viral: A review of replication-selective oncolytic adenoviruses. Oncotarget.

[32] Anderson W.F. (1984). Prospects for human gene therapy. Science.

[33] Bates E.A., Lovatt C., Plein A.R., Davies J.A., Siebzehnrubl F.A., Parker A.L. (2023). Engineering Adenoviral Vectors with Improved GBM Selectivity. Viruses.

[34] Srinivasan V.M., Lang F.F., Kan P. (2021). Intraarterial delivery of virotherapy for glioblastoma. Neurosurg. Focus.

[35] Lang F.F., Conrad C., Gomez-Manzano C., Yung W.K.A., Sawaya R., Weinberg J.S., Prabhu S.S., Rao G., Fuller G.N., Aldape K.D. (2018). Phase I Study of DNX-2401 (Delta-24-RGD) Oncolytic Adenovirus: Replication and Immunotherapeutic Effects in Recurrent Malignant Glioma. J. Clin. Oncol..

[36] Wherry E.J., Kurachi M. (2015). Molecular and cellular insights into T cell exhaustion. Nat. Rev. Immunol..

[37] Fourcade J., Sun Z., Benallaoua M., Guillaume P., Luescher I.F., Sander C., Kirkwood J.M., Kuchroo V., Zarour H.M. (2010). Upregulation of Tim-3 and PD-1 expression is associated with tumor antigen–specific CD8+ T cell dysfunction in melanoma patients. J. Exp. Med..

[38] Jin H.-T., Anderson A.C., Tan W.G., West E.E., Ha S.-J., Araki K., Freeman G.J., Kuchroo V.K., Ahmed R. (2010). Cooperation of Tim-3 and PD-1 in CD8 T-cell exhaustion during chronic viral infection. Proc. Natl. Acad. Sci. USA.

[39] Anderson A.C. (2014). Tim-3: An emerging target in the cancer immunotherapy landscape. Cancer Immunol. Res..

[40] Kiyokawa J., Kawamura Y., Ghouse S.M., Acar S., Barcin E., Martinez-Quintanilla J., Martuza R.L., Alemany R., Rabkin S.D., Shah K. (2021). Modification of Extracellular Matrix Enhances Oncolytic Adenovirus Immunotherapy in Glioblastoma. Clin. Cancer Res..

[41] Liu J., Piranlioglu R., Ye F., Shu K., Lei T., Nakashima H. (2023). Immunosuppressive cells in oncolytic virotherapy for glioma: Challenges and solutions. Front. Cell. Infect. Microbiol..

[42] Maggs L., Cattaneo G., Dal A.E., Moghaddam A.S., Ferrone S. (2021). CAR T Cell-Based Immunotherapy for the Treatment of Glioblastoma. Front. Neurosci..

[43] Wang G., Zhang Z., Zhong K., Wang Z., Yang N., Tang X., Li H., Lu Q., Wu Z., Yuan B. (2023). CXCL11-armed oncolytic adenoviruses enhance CAR-T cell therapeutic efficacy and reprogram tumor microenvironment in glioblastoma. Mol. Ther..

[44] Gesundheit B., Ben-David E., Posen Y., Ellis R., Wollmann G., Schneider E.M., Aigner K., Brauns L., Nesselhut T., Ackva I. (2020). Effective treatment of glioblastoma multiforme with oncolytic virotherapy: A case-series. Front. Oncol..

[45] Kline C., Felton E., Allen I.E., Tahir P., Mueller S. (2018). Survival outcomes in pediatric recurrent high-grade glioma: Results of a 20-year systematic review and meta-analysis. J. Neurooncol..

[46] Martinez-Velez N., Garcia-Moure M., Marigil M., Gonzalez-Huarriz M., Puigdelloses M., Gallego Perez-Larraya J., Zalacain M., Marrodan L., Varela-Guruceaga M., Laspidea V. (2019). The oncolytic virus Delta-24-RGD elicits an antitumor effect in pediatric glioma and DIPG mouse models. Nat. Commun..

[47] Gállego Pérez-Larraya J., Garcia-Moure M., Labiano S., Patiño-García A., Dobbs J., Gonzalez-Huarriz M., Zalacain M., Marrodan L., Martinez-Velez N., Puigdelloses M. (2022). Oncolytic DNX-2401 Virus for Pediatric Diffuse Intrinsic Pontine Glioma. N. Engl. J. Med..

[48] Cooney T., Lane A., Bartels U., Bouffet E., Goldman S., Leary S.E.S., Foreman N.K., Packer R.J., Broniscer A., Minturn J.E. (2017). Contemporary survival endpoints: An International Diffuse Intrinsic Pontine Glioma Registry study. Neuro Oncol..

[49] Ji N., Weng D., Liu C., Gu Z., Chen S., Guo Y., Fan Z., Wang X., Chen J., Zhao Y. (2016). Adenovirus-mediated delivery of herpes simplex virus thymidine kinase administration improves outcome of recurrent high-grade glioma. Oncotarget.

[50] Kang K.D., Bernstock J.D., Totsch S.K., Gary S.E., Rocco A., Nan L., Li R., Etminan T., Han X., Beierle E.A. (2022). Safety and Efficacy of Intraventricular Immunovirotherapy with Oncolytic HSV-1 for CNS Cancers. Clin. Cancer Res..

